# HRQoL impact of stressful life events in children beginning primary school: results of a prospective study in Poland

**DOI:** 10.1007/s11136-016-1371-x

**Published:** 2016-07-27

**Authors:** Maria Kaczmarek, Sylwia Trambacz-Oleszak

**Affiliations:** Department of Human Biological Development, Institute of Anthropology, Faculty of Biology, Adam Mickiewicz University, Poznań, Umultowska 89, 61-614 Poznan, Poland

**Keywords:** Young children, Stressful life events, Stress-related symptoms, Total HRQoL, LGC model

## Abstract

**Purpose:**

To evaluate the relationship between recent stressful life events (SLEs), stress-related symptoms (SRSs), and health-related quality of life (HRQoL) in children beginning primary school.

**Methods:**

A community-based sample of 6- to 8-year-old children (176 boys and 175 girls at baseline) participated in a prospective longitudinal study with three waves of data collection and 1-year interval between subsequent surveys, conducted in the Wielkopolska Province, Poland. Main exposures included nine recent stressful life events and psychosomatic and behavioural symptoms related to stress (SRSs), both self-reported by children. The outcome was total HRQoL assessed by a Polish version of the PedsQL™ 4.0 (Pediatric Quality of Life Inventory) Generic Core Scales questionnaire, 5- to 7-year-old version. To evaluate the relationship between total HRQoL and predictor variables, a latent growth curve (LGC) model using multiple group design (boys and girls) with three waves and two time-varying covariates, the SLEs and SRSs, was applied.

**Results:**

An unconditional multi-group LGC model revealed that the total HRQoL changed over time in a linear trajectory. After incorporating to the model, two time-varying covariates, SLEs and SRSs, the first predictor for HRQoL was only significant at the last wave in girls and at two subsequent waves, except for baseline, in boys. The second predictor revealed significant negative impacts on HRQoL over the entire period of time in both boys and girls suggesting that the pathway underlying the association of SLEs with HRQoL may be mediated by SRSs. Mean values of HRQoL at each time points did not show gender differences.

**Conclusions:**

The findings of the present study may help to develop and implement a health and safety protection training programmes addressed to parents, caregivers, and practitioners to make children’s lives easier.

## Introduction

It is well documented that childhood exposure to certain potentially stressful life events (SLEs) put them at a higher risk of distress (negative stress) which then may increase the risk of *adverse health outcomes* and poor quality of life [1, 2]. Children of all ages experience stressful life events, but the type and the way a child or youth express their persisting stress *depend* on their age, gender, level of development, and the individual’s threshold for responses to stressful stimuli [3]. For younger children, divorce/marital conflict, entry into the school and beginning of a new school year, negative peer pressure, school failure, death of a loved one, a move to new home, natural disasters, and chronic illness mean new challenges that the child must cope with. In these children, stress can manifest itself through somatic complaints such as headache, stomach ache, abdominal and other pains, sweating, nausea and vomiting, muscle stiffness, and others [4]. Common changes can also include behavioural disturbances such as acting irritable or moody, displaying angry or aggressive outbursts, expressing worries, sleeping too much or too little, eating too much or too little, and having impaired attention and concentration [5]. Repeated stressful situations put a strain on the body that may have an effect on the altered immune function and generally is linked to *worsening* the course of chronic medical conditions [6–8], and multiple physical and mental health problems in childhood [9] and during the subsequent stages of life [10–12].

**Fig. 1:**
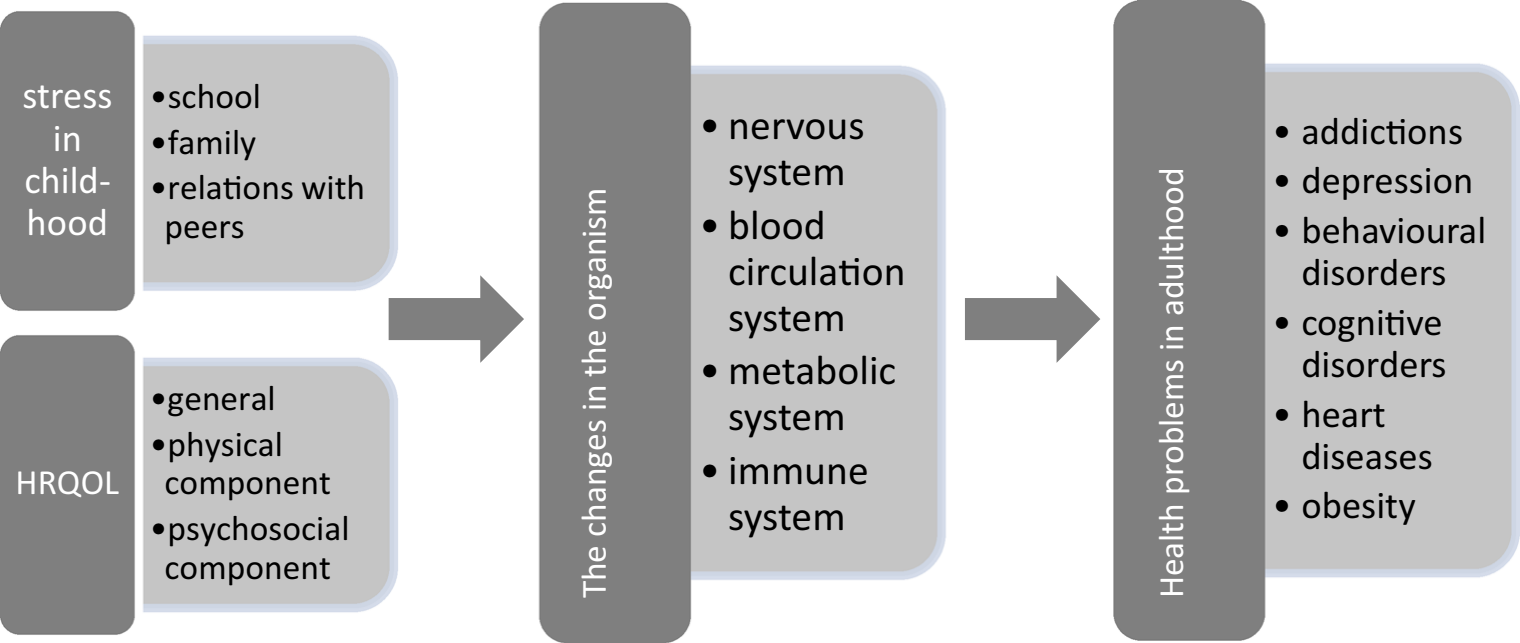
Effects of childhood stress on health problems later in life. Modified after Underdown [10], Middlebrooks and Audage [11], Marin et al. [12]

The long-term physical health outcomes include heart disease, stroke, cancer, gastrointestinal problems, diabetes, and others [13]. Although the exact biological mechanism by which SLEs may cause multiple comorbid conditions remains to be elucidated, recent evidence suggests that physiological changes might be a potential mediator between childhood stressors and stress-induced illnesses later in life [14] (Fig. 1).

There are numerous studies providing evidence of inverse association between exposure to traumatic events and HRQoL in children. Using a 23-item Pediatric Quality of Life Inventory (PedsQL), Coker et al. [15], in a sample of 5,147 fifth grade students and their parents, found a significant association between the number of recent family-related SLEs experienced by a child and lower HRQoL; children who experienced more family-related SLEs were more likely than their counterparts to have worse HRQoL both for the total and for the physical and psychosocial components of the measure.

Moderate effect with no gender differences of recent SLEs on HRQoL were found in a representative sample (840 subjects at baseline) of Spanish adolescents who completed the KIDSCREEN-27 to measure HRQoL [16].

Another study on 170 preschool children, aged 3 through 5 years, revealed that children who had been exposed to 1–3 and 4 or more types of SLEs had significantly worse HRQoL and psychosocial health than children not exposed to SLEs [17]. However, the reviewed studies are cross-sectional and may not help us understand statistical associations between SLEs and HRQoL in causal terms.

Recent multilevel analysis of generic HRQoL in children and adolescents aged 8–18 years conducted in 12 European countries with KIDSCREEN-52 has revealed significant HRQoL variability across countries [18]. A key finding from this study was the importance of social and cultural contexts for the health and well-being in childhood and adolescence. This conclusion has encouraged us to undertake a systematic study on the childhood HRQoL in Poland with focus on its impacts of stressful life events. There is a significant gap of knowledge about HRQoL of younger children in Poland. Everything that we have learned so far considers adolescents, those 11-, 13- and 15-year-old participating in the Health Behaviour in School-aged Children (HBSC) study [19] and those 10–18 years old, participating in the ADOPOLNOR study [20]. These two cross-sectional studies have provided interesting findings on social and cultural covariates of HRQoL but not on SLEs.

Several studies have also shown that for younger children, one of the most frequently reported stressful life events is the entry into the school and beginning a new school year [1, 21]. The transition from home to school is believed to be a major milestone in the lives of children who share their lives between home and school—new social environment since then [22–24]. As from 2014, the compulsory starting age for primary education in Poland is six [25], we have decided to pay our attention to children who turned 6 years of age and began primary school attendance. In this way, we decided to fill the above-mentioned knowledge gaps.

**Fig. 2:**
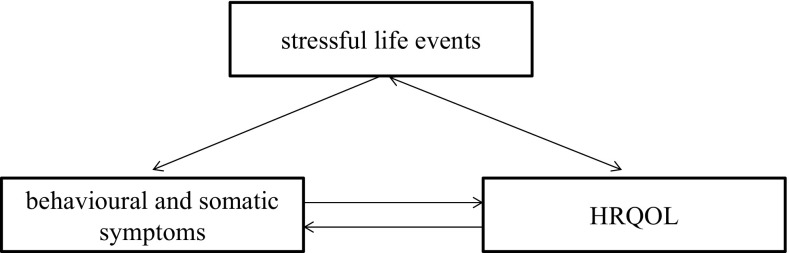
Conceptual framework for the present study on health-related quality of life in the early primary school-age children 6–8 years

Assuming that recent SLEs, defined as personal, familial, and school life changes that induce stress and stress-related somatic and behavioural symptoms are associated with reduced HRQoL, the present study aims to assess the influence of recent SLEs and stress-related symptoms (SRSs) on HRQoL of 6-year-old children and to observe changes during the 3-year period of study. The conceptual framework for this study is shown in Fig. 2.

We hypothesized that (1) there exists substantial change in HRQoL over time, and there is substantial variability between children in their HRQoL trajectories, (2) recent experience of SLEs is the major factor driving the decline in children’s HRQoL over time, and SRSs may modulate this effect, (3) there are differences in HRQoL trajectory between boys and girls. The research questions of this study are as follows: (1) What is the pattern of HRQoL changes over time? (2) Are SLEs and SRSs associated with systematic decline in HRQoL? (3) Do boys and girls differ in their HRQoL trajectories?

## Materials and methods

### Study design and sampling procedure

A longitudinal study was carried out from October 2011 to October 2013 with three waves of data collection on a randomly selected sample of children aged 6 years at baseline, students of the zero grade of primary schools in the Wielkopolska Province and its capital, the city of Poznań, Poland. Interviews were conducted once a year, always in autumn—in the first month of a new school year).

Sampling procedure was a stratified two-stage cluster sample design done primarily for the research project ADOPOLNOR and described in details elsewhere [26, 27]. For the first sampling stage, schools were sampled from the sampling frame provided by the Ministry of Education for the Wielkopolska Province via the Poznań Board of Education and stratified by rural and urban areas. In this way, out of 1217 primary schools, 25 schools were selected, and for the purposes of this study, eight schools located in Poznań (large city), and 12 in the Wielkopolska Province (small-to-medium-sized cities and rural areas) were selected.

Sample size was calculated using the formula for longitudinal study design with the attrition [28]. The number of selected subjects was 325. In order to prevent sample loss, this number was increased to 351. Thus, sample size at baseline was 351 (176 boys and 175 girls). Due to attrition, the sample size decreased to 311 (157 boys and 154 girls) and to 291 (140 boys and 151 girls) in the second and third time points of the study, respectively. Of the initial 351 children, 291 (82.9 %) had a complete valid dataset.

Design and study protocol was approved by the Bioethics Commission of the Poznań University of Medical Sciences and the Poznań Board of Education. All examinations were performed in compliance with principles outlined in the Helsinki Declaration and subsequent amendments. Parents gave a written consent for their children to participate in the study.

The study criteria for eligibility were: a chronological 6 years, attending primary school grade 0, no physical or intellectual disability, no medical records of acute or chronic conditions.

Examinations were performed in school nursery rooms during morning hours always by the same researcher (ST-O), who individually interviewed each child. The interview schedule was carefully designed to be comprehensive and as unbiased as possible. During the interview, children were asked to point out emoticons: smiley (never a problem), normal (sometimes a problem), and sad (almost always a problem) faces in response to interview questions.

### Measures

Data on pregnancy history and child’s health profile were collected by a questionnaire administered to parents.

Standing body height was measured according to standard procedures with a portable Swiss-made GPM anthropometer to the nearest 1 mm [29]. Body weight was measured to the nearest 0.1 kg on a calibrated electronic scale (Precision Health Scale). Body mass index (BMI) was calculated by taking a subject’s weight (kg) and dividing it by his/her height squared (m^2^).

Stressful life events were self-reported by children and measured using a self-report checklist of traumatic events that had occurred in the last month. The SLEs inventory consisted of nine items, which were selected from the Coddington’s Life Events Scale (CLES—P) to represent the most frequently reported events by children under the age of 10 years: death of a close relative or a pet, accident/injury/illness of the subject, parental separation/divorce, moving the whole/a member family, birth of a new brother or sister, natural disaster, changing school/class, being bullied at school, any other stressful event as indicated by the subject [30]. The number of SLEs was counted per child at each wave.

Stress-related somatic and behavioural symptoms were self-reported by children and measured using a self-report checklist of somatic (stomach ache, headache, nausea and vomiting, diarrhoea, sweating, dizziness, pains, sleeplessness muscle stiffness, and nervousness) and behavioural (tantrums, quarrels/fights, nail biting, thumb sucking, sleep disturbance, crying, irritability) disorders that had occurred during the last month in reaction to stressful stimuli [4]. The number of SRSs was counted per child at each wave and a combined score of somatic and behavioural symptoms was taken to further analyses.

HRQoL of children was assessed by a Polish version of the PedsQL™ 4.0 (Pediatric Quality of Life Inventory) Generic Core Scales questionnaire, 5- to 7-year-old version. This questionnaire consists of 23 items and contains four scales that assess the physical, emotional, social, and school dimensions [31–34]. Respondents are asked how much of a problem each item has been during the past 1 month. A Likert scale of three response options (never, sometimes, and almost always a problem) was used, with higher scores indicating better HRQoL. The scores for each of the dimensions as well as the final score are within the range of 0–100 points (the higher the score, the better the HRQoL).

### Data analysis

Children’s chronological age was calculated in decimal values by subtracting the date of examination from the date of birth.

Latent growth curve modelling (LGM), one of the approaches within the framework of structural equation modelling (SEM), was used to answer research questions. The LGM method allows the estimation of both inter-individual and intra-individual differences in patterns of changes for trait(s) over time by creating growth (regression) curve(s), latent growth curve (LGC), on the basis of observed trait at *different time* points *and under the assumption* of its normal distribution [35]. Growth parameters are specified as latent variables, with an intercept representing the score on the outcome variable at an initial time point (at baseline) and a slope describing rate of *change over time* [36, 37]*. For both the intercept and slope, there is a mean and a variance. The intercept mean indicates an* average baseline value, and intercept variance indicates between-individual variability at baseline, whereas the slope mean indicates an average linear rate of change over time and slope variance indicates between-individual variability in rate of changes [38]. SEM is more likely used to determine whether a certain model is valid than to find a suitable model and as such is rather confirmatory than exploratory method [35]. To evaluate the model fit, Chi-square tests and goodness-of-fit indices such as Comparative Fit Index (CFI), Normed Fit Index (NFI), Incremental Fit Index (IFI), and Root Mean Square Error of Approximation (RMSEA) can be applied [39]. The value of CFI NFI and IFI should be more than 0.95 and that of RMSEA less than 0.05 for a model that fits well [39].

In the present study, the total HRQoL score was dependent variable, SLEs and SRSs were two time-varying covariates (explanatory variables) modelled as predictors of HRQoL [40]. Measurements were taken in children aged 6 years at baseline, at three points of time with 1-year interval between subsequent surveys. The skewness and kurtosis of all observed variables but SRSs well supported the assumption of normal distribution of the data. The SRSs data had right-skewed distribution and, therefore, were log transformed prior to analysis.

A univariate, multi-group (boys and girls) unconditional LGC model was first fit for HRQoL data to ascertain whether the growth pattern was linear. Once the linear growth trajectory in HRQoL was confirmed, the SLEs and SRSs were incorporated into the model as time-varying covariates (TVCs). More specifically, at each assessment point, both SLEs and SRSs were modelled as predictors of the time-specific HRQoL measure, in order to examine whether these impacts were significant at each wave, over and above the general linear trend in HRQoL. In both, unconditional and conditional approaches, factor loadings of the intercept parameter to all three observations of HRQoL were fixed to 1. The values 0, 1, and 2, respectively, were assigned to the linear slope parameters to indicate period of 1 year between each of the three survey time points.

Statistical analyses were conducted using the Predictive Analytics Software (PASW Statistics) version 23 IBM SPSS, Chicago. In the modelling and testing of LGC models, the SPSS AMOS version 23 IBM SPSS, Chicago software for the SEM was applied [41]. Maximum likelihood estimation (MLE) was used to avoid bias of missing data and to obtain the output parameters. Chi-square tests, NFI, IFI, CFI, and RMSEA were used to assess the goodness of model fit.

Statistical significance of differences in means was tested using the *F*-test in the one-way ANOVA and additional information on which means were significantly different from each other were obtained using post hoc Scheffe’s test [42]. Chi-square test was used for assessing the *difference* between *categorical variables* [43]*. Statistical tests used in the present study were* comprised of two-way determinations. A value of *p* < 0.05 was considered statistically significant [44]. The effect size of observed differences was calculated using Cohen’s guidelines and expressed in terms of Hedges’ *g* for groups with different sample size. Following benchmarks were used: small effect: 0.20 ≤ *g* < 0.50, medium effect: 0.50 ≤ *g* < 0.80, and large effect: *g* ≥ 0.80 [45].

## Results

The study sample characteristics are summarized in Table 1. Of the 351 child subjects at baseline (mean age 6.58 ± 0.42), 50.2 % were male, and 49.2 % were female, and this male to female proportion remained fairly constant across subsequent examinations. The sample attrition did not either affect the proportion of children from large city to those from other settings. Proportion of children, who experienced SLEs exposure versus those who did not, was similar over the 3-year period. From about 22 to 26 % of children reported no SLEs, more than 60 % of children had experienced one to three SLEs, and 8–11 % had experienced four and more SLEs. Unlike SLEs, the proportion of children who experienced SRSs significantly increased over time and those who experienced four and more SRSs accounted for 31.9 % at baseline and 40.4 % by the end of the study.

**Table 1:** Participant characteristics

Characteristics	Baseline *n* = 351 School grade 0		1st follow-up *n* = 311 School grade 1		2nd follow-up *n* = 291 School grade 2		*p*
	*n* (%)	Mean ± SD	*n* (%)	Mean ± SD	*n* (%)	Mean ± SD	
Age (years)		6.58 ± 0.42		7.46 ± 0.51		8.26 ± 0.42	<0.001
Gender							NS^a^
Boys	176 (50.2)		157 (50.5)		140 (48.1)		
Girls	175 (49.8)		154 (49.5)		151 (51.9)		
Height (cm)		119.9 ± 5.4		125.6 ± 5.9		130.2 ± 6.0	<0.001
Weight (kg)		22.9 ± 4.9		26.7 ± 5.7		29.8 ± 6.7	<0.001
BMI (kg/m^2^)		15.9 ± 2.7		16.8 ± 2.6		17.4 ± 2.9	<0.001
Stressful life events (SLEs)^b^							NS
0	77 (21.9)		83 (26.6)		74 (25.3)		
1–3	235 (66.9)		201 (64.6)		194 (66.7)		
4+	39 (11.2)		27 (8.8)		23 (8.0)		
Stress-related somatic and behavioural symptoms (SRSs)^c^							<0.001
0	54 (15.4)		51 (16.3)		41 (14.1)		
1–3	185 (52.7)		178 (57.3)		132 (45.5)		
4+	112 (31.9)		82 (26.4)		118 (40.4)		
Place of residence							NS
Poznań^d^	141 (40.2)		124 (39.9)		125 (42.9)		
Province^e^	210 (59.8)		187 (60.1)		166 (57.1)		

^a^NS, statistically not significant

^b^Cronbach’s *α* for SLEs = 0.746

^c^Cronbach’s *α* for SRSs = 0.739

^d^Large city

^e^Medium to small-sized cities and rural areas combined

**Fig. 3:**
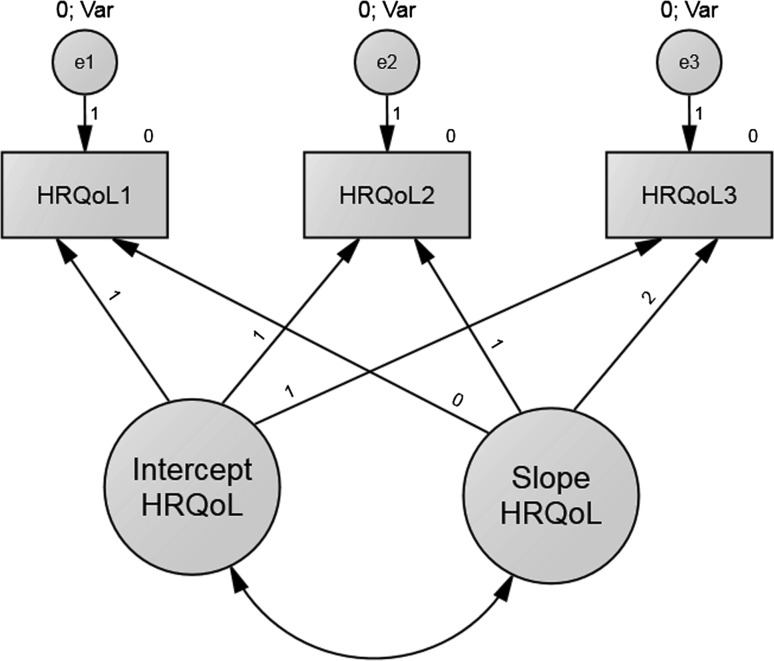
Unconditional LGC model depicting linear growth of the HRQoL over time in the early primary school children aged from 6 to 8 years

The growth pattern of the total HRQoL at ages 6, 7, and 8 is shown in Fig. 3. An unconditional multi-group LGC model showed good model fit: *χ* ^2^ = 25.147, *df* = 7, *p* < 0.001; NFI = 0.964, IFI = 0.979, CFI = 0.977, RMSEA = 0.068, suggesting that the HRQoL changed in a linear trajectory.

As shown in Table 2, the mean value of the intercept of the HRQoL was 74.92 in boys and 76.83 in girls (*p* < 0.001) suggesting that the average initial value of the HRQoL was significantly different from its final value. As shown by the mean value of the slope, there was a linear increase over time on average. The initial HRQoL score was expected to increase on average by 1.11 (*p* = 0.05) scores in boys and by 0.51 (*p* = 0.42) scores in girls in each studied year. The average yearly increase in HRQoL made significant contribution in the corresponding HRQoL score in boys but not in girls. In addition, the variance of the intercept (*p* < 0.001) was statistically significant in both genders indicating significant individual differences in HRQoL at baseline at age 6. The variance of the linear slope was 5.99 (*p* = 0.39) in boys and 15.81 in girls (*p* = 0.04), suggesting that the slopes for individual cases in boys were more often parallel than in girls. It also suggests that in girls, the rate of HRQoL trajectories significantly differed over time. Significant negative covariance between the intercept and slope was found in both genders (−21.94, *p* = 0.04 and −24.40, *p* = 0.04 for boys and girls, respectively), suggesting that higher initial HRQoL would be associated with lower rate of changes in HRQoL over time. In addition, this inverse association was confirmed by correlation coefficient of *r* = −0.80 in boys and *r* = −0.57 in girls.

**Table 2:** Parameter estimates for unconditional LGC model of the HRQoL changes over time in boys and girls aged from 6 to 8 years

Parameter	Estimate	S.E.	C.R.	*p* value
Boys				
Growth parameters				
Intercept mean	74.92	1.15	65.21	<0.001
Slope mean	1.14	0.59	1.92	0.050
Intercept variance	125.12	23.57	5.31	<0.001
Slope variance	5.99	6.92	0.86	0.387
Intercept ↔ Slope covariance	−21.94	10.53	−2.08	0.037
Intercept ↔ Slope correlation	−0.80			
Girls				
Growth parameters				
Intercept mean	76.83	1.13	67.88	<0.001
Slope mean	0.51	0.63	0.80	0.424
Intercept variance	117.68	22.77	5.17	<0.001
Slope variance	15.81	7.66	2.06	0.039
Intercept ↔ Slope covariance	−24.40	10.86	−2.25	0.025
Intercept ↔ Slope correlation	−0.57			

Model fit: boys *n* = 176, girls *n* = 175, *χ* ^2^ = 25.147, *df* = 7, *p* < 0.001, NFI = 0.964, IFI = 0.979, CFI = 0.977, RMSEA = 0.068

**Fig. 4:**
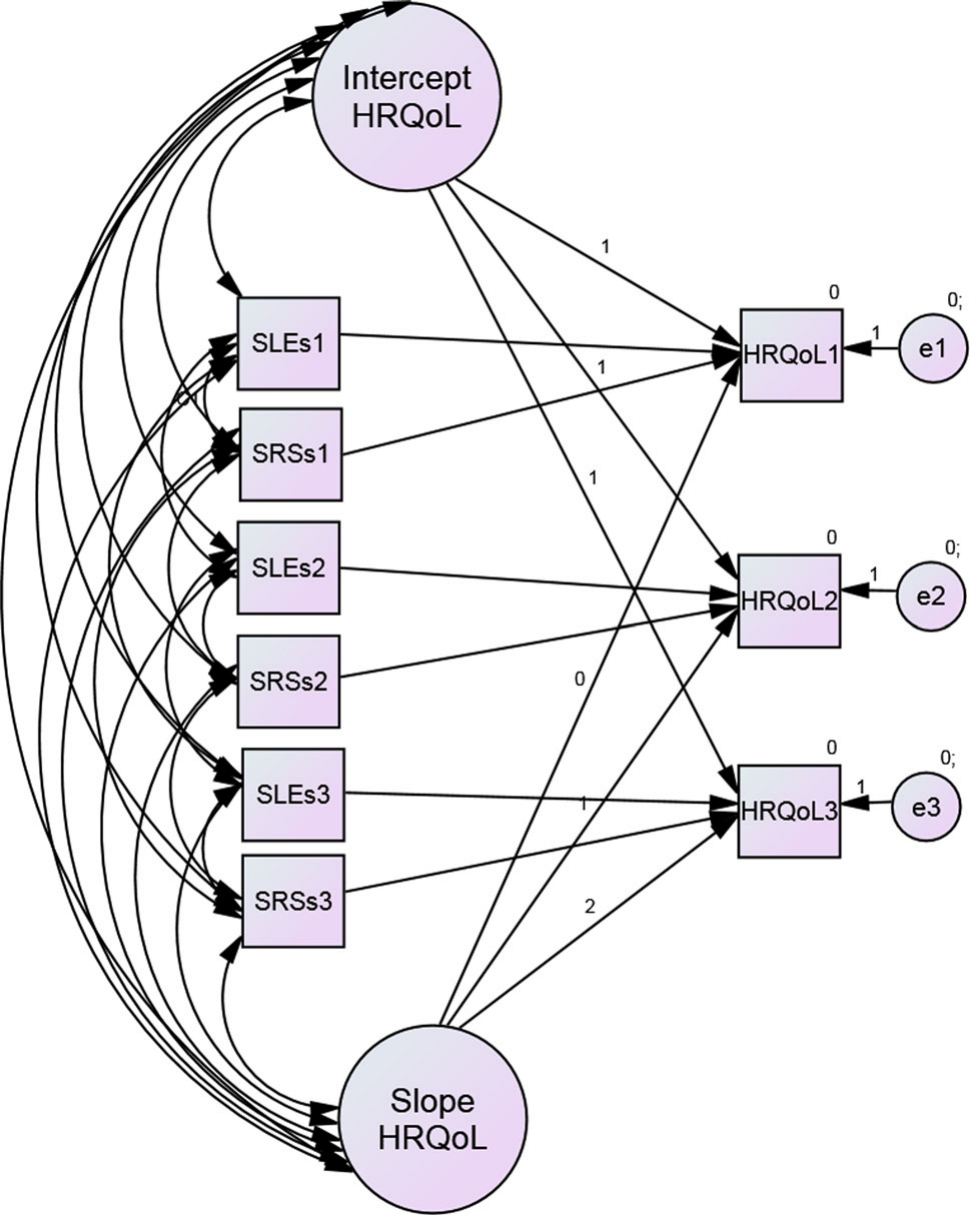
LGC model of the HRQoL trajectory over time in the early primary school children aged from 6 to 8 years with time-varying predictors: stressful life events (SLEs) and stress-related somatic and behavioural symptoms (SRSs)

A multi-group LGC model with two time-varying covariates is shown in Fig. 4. This model resulted in very good fit indices: *χ* ^2^ = 25.267, *df* = 15, *p* = 0.043, NFI = 0.967, IFI = 0.986, CFI = 0.985, RMSEA = 0.048.

Table 3 presents the impact of SLEs and SRSs on HRQoL over time in boys and girls. An average number of SLEs revealed significant decrease and that of SRSs increase over time in both boys and girls (all *p* < 0.001). The two predictors were significantly and consistently positively associated to each other at each time point. Individuals with greater number of SLEs were likely to respond to this exposure with greater number of SRSs.

**Table 3:** Parameter estimates of the proposed latent growth curve (LGC) model for boys’ and girls’ HRQoL trajectories after controlling for the effect of the time-varying covariates

Parameter	Estimate	S.E.	C.R.	*p* value	Estimate	S.E.	C.R.	*p* value
	Boys				Girls			
Growth parameters								
Intercept mean	82.26	3.18	25.90	<0.001	80.96	3.47	23.36	<0.001
Slope mean	4.38	2.69	1.63	0.104	0.99	3.03	0.33	0.743
Intercept variance	32.16	20.84	1.54	0.123	73.20	28.26	2.59	0.010
Slope variance	3.03	12.63	0.24	0.810	17.64	12.63	1.39	0.163
Intercept ↔ Slope covariance	3.98	13.25	0.30	0.764	−19.79	16.13	−1.23	0.220
Intercept ↔ Slope correlation	0.40				−0.55			
Time-varying covariates								
SLEs1 mean (variance)	1.92 (1.36)	0.10 (0.17)	19.01 (8.16)	<0.001	2.11 (1.64)	0.11 (0.20)	19.11 (8.21)	<0.001
SLEs2 mean (variance)	1.73 (1.60)	0.10 (0.20)	17.93 (8.12)	<0.001	1.75 (1.39)	0.10 (0.17)	16.79 (7.95)	<0.001
SLEs3 mean (variance)	1.66 (1.21)	0.11 (0.15)	15.27 (8.03)	<0.001	1.51 (1.00)	0.08 (0.12)	18.03 (8.42)	<0.001
SRSs1 mean (variance)	2.39 (3.00)	0.16 (0.36)	14.52 (8.24)	<0.001	2.66 (3.082	0.16 (0.46)	14.52 (8.34)	<0.001
SRSs2 mean (variance)	2.47 (3.76)	0.15 (0.46)	16.69 (8.21)	<0.001	2.71 (3.60)	0.16 (0.45)	16.48 (8.07)	<0.001
SRSs3 mean (variance)	2.77 (4.35)	0.18 (0.54)	15.28 (8.10)	<0.001	3.01 (3.97)	0.17 (0.47)	18.09 (8.47)	<0.001
SLEs1 ↔ SRSs1 covariance	0.55	0.12	4.41	<0.001	0.53	0.12	4.32	<0.001
SLEs2 ↔ SRSs2 covariance	0.44	0.18	2.50	0.017	0.39	0.17	2.32	0.020
SLEs3 ↔ SRSs3 covariance	0.50	0.18	2.81	0.005	0.31	0.14	2.19	0.028
Standardized path coefficients, *β*								
SLESs1 → HRQoL1	0.07	1.56	0.04	0.561	0.09	1.46	0.06	0.629
SLESs2 → HRQoL2	−0.21	0.78	−0.27	0.010	−0.11	0.82	−0.13	0.173
SLESs3 → HRQoL3	−0.45	1.63	−0.28	0.004	−0.30	0.84	−0.36	0.021
SRSs1 → HRQoL1	−0.43	1.18	−0.36	0.002	−0.32	0.93	−0.35	0.003
SRSs2 → HRQoL2	−0.44	0.51	−0.87	<0.001	−0.33	0.57	−0.58	0.003
SRSs3 → HRQoL3	−0.39	1.00	−0.39	0.030	−0.49	1.06	−0.46	0.005

Model fit: boys *n* = 176, girls *n* = 175, *χ* ^2^ = 25.267, *df* = 15, *p* = 0.043, NFI = 0.967, IFI = 0.986, CFI = 0.985, RMSEA = 0.048

There was gender difference in the pattern of HRQoL impacts of SLEs. In boys, negative significant impact of SLEs on HRQoL was found at largely most time points excluding the baseline (*β* = −0.21, *p* = 0.01 and *β* = −0.45, *p* = 0.004 for the second and third waves, respectively). This means that boys who were exposed to greater number of SLEs through the subsequent period of time were more likely to report lower HRQoL than their counterparts not-exposed to SLEs. However, in girls, SLEs negatively predicted HRQoL at only the last wave of the study (*β* = −0.30, *p* = 0.02) indicating that girls needed longer time of exposure to SLEs to respond in decreased HRQoL score. The same gender pattern of SRSs effects on HRQoL changes over time was found with significant negative associations between these two variables at each time point (*β* = −0.43, *β* = −0.44, *β* = −0.39 and *β* = −0.32, *β* = −0.33, *β* = −0.49 and all statistically significant at *p* < 0.05 at each time point in boys and girls, respectively). Table 4 presents data for further insight into statistical translation of the findings obtained from the model.

**Table 4:** Means and standard deviations of HRQoL in three different groups of stressful life events (SLEs) and stress-related symptoms (SRSs) at three time points, boys and girls combined

PedsQL 4.0	Baseline *n* = 351 School grade 0; Age = 6 years			1st follow-up *n* = 311 School grade 1; Age = 7 years			2nd follow-up *n* = 291 School grade 2; Age = 8 years		
	A1 SLEs = 0 *n* = 77	B1 SLEs = 1–3 *n* = 235	C1 SLEs = 4+ *n* = 39	A2 SLEs = 0 *n* = 83	B2 SLEs = 1–3 *n* = 201	C2 SLEs = 4+ *n* = 27	A3 SLEs = 0 *n* = 74	B3 SLEs = 1–3 *n* = 194	C3 SLEs = 4+ *n* = 23
	Mean ± SD	Mean ± SD	Mean ± SD	Mean ± SD	Mean ± SD	Mean ± SD	Mean ± SD	Mean ± SD	Mean ± SD
Total score	78.8 ± 16.5	74.8 ± 16.1	69.7 ± 15.7	81.5 ± 16.4	75.4 ± 13.2	69.9 ± 16.0	81.4 ± 12.9	77.0 ± 12.0	71.5 ± 11.0
Effect size^a^	A1–B1	B1–C1	A1–C1	A2–B2	B2–C2	A2–C2	A3–B3	B3–C3	A3–C3
Total score	– NS	– NS	0.56**	0.41**	0.37*	0.71**	0.36**	0.46*	0.84**

	A1 SRSs = 0 *n* = 54	B1 SRSs = 1–3 *n* = 185	C1 SRSs = 4+ *n* = 112	A2 SRSs = 0 *n* = 51	B2 SRSs = 1–3 *n* = 178	C2 SRSs = 4+ *n* = 82	A3 SRSs = 0 *n* = 41	B3 SRSs = 1–3 *n* = 132	C3 SRSs = 4+ *n* = 118
	Mean ± SD	Mean ± SD	Mean ± SD	Mean ± SD	Mean ± SD	Mean ± SD	Mean ± SD	Mean ± SD	Mean ± SD
Total score	87.3 ± 13.4	78.3 ± 12.7	64.9 ± 12.7	83.1 ± 11.9	75.5 ± 14.6	69.3 ± 14.4	82.6 ± 13.8	78.8 ± 13.9	70.4 ± 13.9
Effect size^a^	A1–B1	B1–C1	A1–C1	A2–B2	B2–C2	A2–C2	A3–B3	B3–C3	A3–C3
Total score	0.69**	1.05**	1.72**	0.54*	0.43**	1.02**	0.27 NS	0.60**	0.87**

NS, statistically not significant *p* value

Statistically significant at * *p* < 0.05; ** *p* < 0.01

^a^Effect size—benchmark Hedges’ *g* effects: small effect: 0.20 ≤ *g* < 0.50, medium effect: 0.50 ≤ *g* < 0.80, and large effect: *g* ≥ 0.80

Children who were exposed to at least one type of SLEs were likely to have lower HRQoL over time than their peers who were not. The difference was not statistically significant at baseline, but it became significant in the course of the study. The effect size was assessed small. For children in the 4+ SLEs risk group, the effect size of difference in HRQoL between them and their not-exposed counterparts varied from small to large throughout the entire period. For children who experienced four and more SRSs as compared to those without any SRSs, the effect size was assessed to be large across all time points.

## Discussion

This is the first to our knowledge study showing the association of SLEs and SRSs with the level of HRQoL in children from the first grade of primary school onwards. As predicted, the study proved that children exposed to multiple SLEs and experienced multiple SRSs were likely to have impaired HRQoL over the time period. The effect size of the differences between children exposed to SLEs and those not exposed varied from small to large. The effect size of the differences between children who experienced multiple stress-related symptoms and those who did not varied from medium to large. Furthermore, our findings suggest that the likelihood that children who have low HRQoL will catch up their peers with higher level of HRQoL within the first 2 years of primary school seems at best rather doubtful (negative and statistically significant values of path coefficients from SLEs and SRSs to HRQoL). The findings of this study also suggest that pathways by which the SLEs have a prolonged impact on children’s HRQoL may be through psychosomatic and behavioural reactivity to the stressors.

Additionally, anthropometric indicators of physical growth such as body height, body weight, and BMI proved that children were growing normally within the reference values typical for their peers within the same chronological age cohort [46].

The findings of the present study corroborate well with those presented by Coker et al. [15] who studied the association between occurrence of multiple family-related SLEs and the level in HRQoL in the fifth grade students. They found that children with four or more SLEs had greater odds of impaired HRQoL than children without any SLEs.

*Several other studies* have similarly found a linear relationship between experiencing more types of traumatic events with worse HRQoL outcomes in both younger, preschool children [47] and adolescents [48]. The deleterious consequences associated with maltreatment of younger children are substantial for increased risks of maladaptation within the biological, psychological, and social domains of child’s development [49]. Childhood maltreatment is also associated with negative views towards learning and poor school performance [50]. There is a growing body of evidence that children of divorced parents are far more likely to function worse than their peers whose parents did not divorce, having emotional problems, such as anxiety and depression, poor school achievements, and antisocial behaviours [51]. Another study showed that children of alcoholics who were well adapted at age 3–5 years, at later ages, 9–11 and 12–14 years, were more likely to exhibit disruptive *behaviours with greater intensity* than children of parents who do not drink [52].

Studies on migrant preschool children living in Switzerland [53] and in Germany [54] proved that exposure to traumatic stimuli in a new living environment can have a significant negative impact on their HRQoL and well-being.

Our findings revealed that children who were exposed to SLEs were more likely to complain of multiple somatic and behavioural symptoms commonly related to stressors than their not exposed counterparts. These findings are in line with other studies [55–57]. Valizadeh et al. [57] investigating stress symptoms reported by primary school students from Tabriz (Iran) found stomach ache and headache fairly frequently reported by both boys and girls (26.4 vs. 39.1 %; *p* < 0.001 and 44.8 vs. 47.6 %; *p* = 0.44). They also found that girls reported more symptoms in response to stress than boys did.

There are several causal pathways that have been hypothesized for understanding the mechanisms that transfer SLEs to impaired HRQoL on the individual level [58, 59]. Our findings suggest that the association of SLEs with HRQoL may be mediated by stress-related symptoms. The accumulation of these symptoms over time may lead to adverse health outcomes and diseases [2, 10 11]. The rate of this process may significantly vary among children because each child experience negative life events in his/her unique ways and his/her physiological/behavioural responses to these events will be unique as well. The psychophysiological nature of the human chronic stress responses via the hypothalamic pituitary adrenal (HPA) pathway has well been known from both animal models and human observational studies [60]. There is a growing body of evidence that chronic stress may have an effect on the altered immune function and is generally linked to *worsening* the course of chronic medical conditions [52, 61, 62] and behavioural problems in children [9, 63].

The review of literature data has shown an inconsistency as to the gender differences in the association between SLEs and HRQoL. Villalonga-Olives et al. [17] in their follow-up study of 840/454 Spanish adolescents found a moderate association between recent LEs and HRQoL, and without gender differences in this association. In the above-mentioned studies of Coker et al. [15] and Roberts et al. [16], girls who were exposed to four or more SLEs were less likely to have impaired HRQoL than boys. Our findings revealed that gender difference in mean HRQoL score at each time point was not statistically significant indicating similar level of HRQoL in early primary school children aged from 6 to 8. The pattern of growth trajectory was only slightly different in girls than boys in that girls varied significantly in the rate of growth with an average yearly increase which did not significantly contributed in the final score of HRQoL. Another difference was that after controlling total HRQoL for SLEs and SRSs, the first predictor was not significant across two waves in girls, whereas in boys only at baseline.

### Study strengths and weaknesses

There are several strong and weak points that should be considered when interpreting results of this study. First, this study provides some of the first published data on the HRQoL burden affected by SLEs and SRSs among primary school children, aged 6–8 years. Second, this study was designed as a community-based, longitudinal study. Such a design allows researcher to analyse the association between time-varying outcome and explanatory variables and ignore intra-individual variation bias as the age-adjusted individual variation remained on the same level over the course of study period [64]. Third, children were interviewed by the same person, a highly trained investigator who could supervise them self-reporting their subjective feelings.

There are, however, some limitations derived from a longitudinal design. There is always a loss of study participants. In our study, the cumulative attrition rate accounted for 17.1 % and as such met data quality requirements. Other possible sources of bias include the inherent biases of self-reported data as children have difficulties in identifying and verbalizing their feelings. To reduce this potential bias, an instrument with good psychometric properties, the PedsQL was administered to children, and children used emoticons to answer interviewer’s queries.

Another study limitation is that the Polish version of the PedsQL, although widely used in clinical practice and research studies, has not been validated in young children yet. Considering all these limitations, the results of the present study, although stimulated, should be interpreted with caution.

## Conclusions

This study provides evidence for the negative impact of recent stressful life events and stress-related symptoms on the total HRQoL in early primary school children aged 6–8. The pathway underlying the association of SLEs with HRQoL may be mediated by SRSs. Exposure to multiple negative SLEs may lead to adverse physical and mental health conditions and maladjustment to the family, school, and social environments.

These findings may have important implications for children’s parents, caregivers, and practitioners working with young children. Parents and caregivers should improve their knowledge, skills, and ability to successfully advocate for the needs of their children. Practitioners need to have positive relationships with parents and caregivers and provide them with knowledge and skills how to reduce harmful stressors from daily life environments and thus enhance children’s quality of life.

## Abbreviations

CFI: Comparative Fit Index
CR: Critical ratio
HRQoL: Health-related quality of life
IFI: Incremental Fit Index
LGM: Latent growth curve modelling
LGC: Latent growth curve
MLE: Maximum likelihood estimation
NFI: Normed Fit Index
PedsQL: Pediatric Quality of Life Inventory
RMSEA: Root mean square error of estimation
SE: Standard error
SEM: Structural equation modelling
SLEs: Stressful life events
SRSs: Stress-related somatic and behavioural symptoms
TVC: Time-varying covariate
